# Antibiotic resistance: calling time on the ‘silent pandemic’

**DOI:** 10.1093/jacamr/dlac016

**Published:** 2022-03-18

**Authors:** Marc Mendelson, Michael Sharland, Mirfin Mpundu

**Affiliations:** 1 Division of Infectious Diseases and HIV Medicine, Department of Medicine, University of Cape Town, Cape Town, South Africa; 2 Institute of Infection and Immunity, St George’s University, London, UK; 3 ReAct Africa, Lusaka, Zambia

## Abstract

It is time to stop referring to the antibiotic resistance pandemic as ‘silent’. Continuing to use such a term denies the reality that antibiotic-resistant bacterial infections, driven by misuse and abuse of antibiotics by humans against microbial ecosystems that we should be living in symbiosis with, is wrong. Both our terminology and who the real ‘enemy’ is in relation to antibiotic resistance demands serious reconsideration.

Words matter, as do the meaning they convey and the aspiration that lies behind them.

In the shadow of COVID-19, antibiotic resistance (ABR), one of the world’s worsening pandemics, has been ‘silenced’. A pandemic that, pre-COVID-19, commanded a high-level meeting of the United Nations General Assembly^1^ (only the fourth health topic to be afforded that recognition) and was top of the health agenda for G7 and G20 meetings,^2,3^ the subject of calls-to-action and a focus area for Ministries of Health from Albania to Zimbabwe, has been sidelined.

De-prioritizing efforts to mitigate antibiotic resistance has enabled yet another respiratory virus—SARS-CoV-2—to drive inappropriate antibiotic use worldwide. Despite a low overall pooled proportion of patients who had laboratory-confirmed bacterial coinfections of 7% (systematic review and meta-analysis of 30 studies, 27 of which were in hospitalized patients),^4^ the International Severe Acute Respiratory and emerging Infection Consortium (ISARIC) clinical database attests to the inordinate amount of misuse and overuse of antibiotics, with 61% of persons admitted to hospital receiving one or more antibiotics.^5^

In response to reduced global attention to ABR, the term ‘the silent pandemic’ has gained traction in publications,^6^ in political speeches^7^ and on social media. But what does that convey to those who we are trying to encourage to join the efforts to save antibiotic efficacy for future generations? We believe that most people have adopted this term to convey comparison with the very ‘loud’ COVID-19 pandemic and its prioritization.

However, something that is labelled as ‘silent’ is often kept so. Although said outside of the ABR context, Sir Thomas More’s quote, ‘*qui tacet consentire videtur* [he who is silent seems to consent]’, is apt. By propagating the use of the term ‘silent pandemic’ we are at risk of encouraging the very people we want to galvanize to action to consent silently to the status quo.

COVID-19 has reminded us of the power that threat has as a driver for action, be it behaviour change (in adopting public health interventions to stop transmission) or designating unprecedented funds to research and development of new antivirals, vaccines and diagnostics. By conveying ABR as a silent pandemic, are we tacitly propagating that silence when we should be espousing completely the opposite?

If we are to ramp up the ABR decibels, then we need to (i) speak to truth and (ii) understand what people relate to. For far too long, we have exhorted people to join the ‘fight’ against antibiotic-resistant bacteria; our fight is not with bacteria, it’s with humans. It is us who overuse and misuse antibiotics, us who feed antibiotics to animals for food production, and us who pollute the environment through antibiotic manufacturing and other means as far-fetched as incorporating antibiotics into antifouling paint to prevent barnacle attachment on ships.^8^

Climate change and emerging infectious diseases have focused attention on the importance of protecting the environment. This is equally relevant to ABR, and it affords us an opportunity to focus on a relatable concept—the destruction of our own body’s environment when we abuse antibiotics. As symbionts, living an interdependent relationship with bacteria and other microbes, which collectively outnumber our own human cells,^9^ we have an opportunity to focus people’s attention on the harm of destroying our own personal environment and the critical role that bacteria and other microbes play in our immune response, in outcompeting pathogens by creating barriers for entry to our body’s surface and internal environment, and in digestion. The importance of this environment to health and disease has been recently reviewed.^10^

If we are to shout from the rooftops to regain the attention that antibiotic resistance deserves, then it’s time, once again, to think more carefully about the terminology we use,^11^ call time on the use of ‘silent’, and point out even more clearly that, as for climate change, humans need to take responsibility for the destruction of a (microbial) environment, which should be protected from antibiotics except when clearly necessary, in order to limit further emergence of bacterial resistance and maintain health through critical human–microbial and animal–microbial symbiotic relationships.
